# Feeding Problems Including Avoidant Restrictive Food Intake Disorder in Young Children With Autism Spectrum Disorder in a Multiethnic Population

**DOI:** 10.3389/fped.2021.780680

**Published:** 2021-12-13

**Authors:** Gudrun Nygren, Petra Linnsand, Jonas Hermansson, Lisa Dinkler, Maria Johansson, Christopher Gillberg

**Affiliations:** ^1^Gillberg Neuropsychiatry Centre, Sahlgrenska Academy, University of Gothenburg, Gothenburg, Sweden; ^2^Child and Adolescent Specialist Centre, SV Hospital Group, Gothenburg, Sweden

**Keywords:** ASD, feeding disorders, ARFID, PFD, early regulatory problems

## Abstract

We examined feeding problems, including Avoidant Restrictive Food Intake Disorder (ARFID), in preschool children with Autism Spectrum Disorder (ASD). Data were collected from a prospective longitudinal study of 46 children with ASD in a multiethnic, low resource area in Gothenburg, Sweden. Feeding problems were found in 76% of the children with ASD, and in 28%, the criteria for ARFID were met. The study highlights early onset age, the heterogeneity of feeding problems, and the need for multidisciplinary assessments in ASD as well as in feeding problems, and also the need for further elaboration of feeding disorder classifications in children.

## Introduction

Problems with eating and feeding in children with Autism Spectrum Disorder (ASD) have been reported to occur in 50–90% of cases (1, 2). Studies have highlighted that feeding problems might show up very early in the course of ASD (3, 4), but research on early feeding disorders in ASD has been limited.

The early presentation of ASD is strongly associated with impairments in social communication and reduced joint attention behaviors, but also with unusual reactions to sensory stimuli (5–9). Sensory, motor and emotional regulation problems might emerge as prodromal signs already in the first year of life (10–12). Eating, sleeping, and excessive crying problems in infancy are often referred as to regulatory problems (RPs) (13, 14) and are the most frequent causes of parental concerns and contact with health services in the general child population (15). Persistent RPs have been pointed out as a risk factor for later developmental problems (15–18). In infants with later diagnosed ASD, an increased incidence of distress reactions, irritability, negative affect, as well as early sleeping problems has been reported (10, 19, 20). In a Swedish study, early RPs were frequent in children who later received a diagnosis of ASD compared to population rates (12).

High rates of coexisting neurodevelopmental and medical conditions, including feeding disorders, in ASD has been highlighted since the 1980s (21, 22), but particularly during the last decade (23–29). In 2010, Gillberg (30) launched the concept of Early Symptomatic Syndromes Eliciting Neurodevelopmental Clinical Examinations (ESSENCE), with a view to highlighting the overlap of symptoms and coexisting neurodevelopmental disorders in early childhood. Feeding problems stand out as one of the early challenging conditions.

*Eating and feeding problems* might in children with ASD, as in all children, be due to and/or connected to different medical causes such as food allergy, gastrointestinal disorders, disorders in oral and nasopharyngeal function, and congenital heart diseases or neurological disorders. Studies have reported a higher prevalence of gastrointestinal diseases, food allergies, and medical co-morbidities in general in children with ASD than in typically developing children (31–33). ADHD and other neurodevelopmental coexisting disorders might also affect feeding. It is reasonable to assume that, in many cases, several factors contribute to the feeding problems. Psychosocial problems in the family, lack of support, and daily routines need to be explored in all cases. Research has also shown that children living in disadvantaged economic groups are at an increased risk of thinness (34) and that parental feeding practices can contribute to common feeding problems in children (35).

Eating and feeding problems range from mild, transient *difficulties* to severe behavioral and /or medical *feeding disorders*. However, there is still no uniform use of terms and definitions for feeding problems or entirely clear diagnostic classifications to cover the complexity of feeding disorders' etiologies and clinical manifestations. In the International Statistical Classification of Diseases and Related Health problems, 10th Revision (ICD-10), the diagnostic codes for pediatric feeding disorder either require an absence of organic disease to code “Feeding disorders of infancy and childhood” (F98.2) or use the unspecific “Feeding difficulties and mismanagement” (R63.3) (36). These two diagnostic codes in ICD-10 have, to our knowledge, most often been used in clinical settings for feeding problems in children with ASD. The ICD-11 categories and criteria were not available at the time of conducting the present study and will not be further discussed here. Recently, in order to comprehensively cover various aspects of feeding disorders, a broader concept of *Pediatric Feeding Disorder (PFD)* has been proposed (37). An interdisciplinary consensus group has, by using the framework of the International Statistical Classification of Functioning, Disability and Health (ICF) (38), defined PFD as “impaired oral intake that is not age-appropriate, and associated with medical, nutritional, feeding skill and/or psychosocial dysfunction”. In a nationwide study, the annual prevalence of PFD in children under five years of age in the US was reported in the range of 2.7–4.4% (39).

In 2013, the diagnosis *Avoidant/Restrictive Food Intake Disorder (ARFID)* was introduced into the 5th Edition of the Diagnostic and Statistical Manual of Mental Disorders (DSM-5). The diagnosis has no age restriction and is manifested as inability to meet nutritional and/or energy needs and leads to at least one of the following: weight loss or failure to achieve appropriate weight gain; nutritional deficiency; dependence on enteral feeding or nutritional supplements; or significant interference with psychosocial functioning (40). The knowledge of clinical characteristics in ARFID is limited. ARFID involves a complex etiology, and it has been suggested that ARFID and other neurodevelopmental disorders are overlapping (41, 42), consistent with the concept of ESSENCE (43). In a recent study by Farag et al. (44), it was found that ARFID was more frequent among younger children (4–9 years) and in children with comorbid ASD. Studies have suggested ARFID include three often overlapping presentations: (1) lack of interest in eating, (2) restrictive eating due to sensory sensitivity, and (3) avoidance of eating because of fear of negative consequences (45–47). Sharp and Stubbs (48) have proposed subtyping ARFID by the age of onset and whether eating difficulties involve volume or variety. The prevalence of ARFID in the general child population is not yet fully known. There have been a few population-based studies, based on self- or parent-reports, presenting heterogeneous results, ranging from 0.3 to 5.5% (49–53) with one outlier reporting 15.5% (51). Since ARFID was introduced in the DSM-5, it has emerged that there are still difficulties in clinical work and research to find common definitions for the diagnostic criteria (e.g., how is weight loss or failure to achieve appropriate weight gain defined in children of different ages). Recently, a workgroup of experts offered some definitions for operationalizing the ARFID criteria to be tested in clinical populations (54).

The aim of the present study was to examine feeding problems in preschool children with ASD. The primary goals were to study (a) the prevalence of feeding problems, (b) the prevalence and characteristics of ARFID within the group, and (c) the onset age and possible prodromal signs for feeding disorders in ASD. In addition to these primary goals, current classifications of feeding disorders in young children were discussed.

## Materials and Methods

Data were collected from an ongoing prospective longitudinal study of young children diagnosed with ASD in an area with a high prevalence of people with multiethnic background and/or socioeconomically disadvantaged situation in Gothenburg, Sweden. Details regarding the multidisciplinary team for both assessments and interventions have been described previously (55, 56). Two of the authors (GN, pediatrician and child psychiatrist and PL, psychologist) had personally examined all included children, met them and their families in interventions over a period of at least two years and re-examined all children in the multiprofessional team at follow-up. For this study, retrospective analyses were performed on collected data.

### Participants

The study group consisted of 46 children (9 girls, 37 boys) born 2010–2016 and diagnosed with DSM-5 ASD (American Psychiatric Association 2013) by the multidisciplinary team. The average age for ASD diagnosis was 38 months (range = 22–59 months, standard deviation (SD) = 9). Regarding ASD severity level, 25 children (54%) had ASD level 1, and 21 (46%) had ASD level 2. Six children (13%) had average intellectual functioning (Intelligence Quotient (IQ)/Developmental Quotient (DQ) ≥85), 12 (26%) borderline intellectual functioning (IQ/DQ 70–84), and 28 (61%) had intellectual disability (IQ/DQ <70). All 46 children also had other coexisting neurodevelopmental conditions, such as sleeping problems, early ADHD symptoms, language disorders, and/or epilepsy. All but one of the children lived in the same area with a low average income, high prevalence of ill health, and a high level of unemployment (57). Most of the parents had non-Swedish background (93%). For more details regarding participants, see Linnsand et al. (56).

The children received interventions in fortnightly sessions in collaboration with the family and preschool staff. For the majority of the children, the interventions were based on the Early Start Denver Model (low intensity due to limited resources) (58). Other treatment efforts were given according to the needs of the individual child (coexisting neurodevelopmental and medical conditions) and the family's needs.

### Data Collection

Data were collected from all available medical records for each child from the perinatal period and birth to six years of age. Two of the authors (GN and PL) independently scrutinized records from the Child Health Center (CHC) and the comprehensive neuropsychiatric assessment. Medical records from other clinics were also collected for the children who had been assessed for any other medical concerns. Data were scrutinized in great detail, including demographics, prenatal and perinatal history, RPs, and other early symptoms, as well as all coexisting conditions, neurodevelopmental and medical, with extra focus on feeding problems. Data for all children included growth curves and body mass index (BMI), blood tests and results from other medical examinations, and required treatment approaches. For more details regarding definitions of demographics, prenatal and perinatal period, see Linnsand et al. (56).

### Measures

#### The Essence-Q

The ESSENCE Questionnaire (ESSENCE-Q) was developed as a brief screener to identify children with neurodevelopmental difficulties who might have neurodevelopmental disorders (30, 59) (Supplement Material 1). It consists of 12 items covering: general development, motor development, sensory reactions, communication/language, activity/impulsivity, attention/concentration, social interaction, behavior, mood, sleep, *feeding*, and “funny spells”/absences. Here, the ESSENCE-Q rating was based on clinical observation of the child and on parent interview at the first visit in the neuropsychiatric assessment when the child was referred with a suspicion of ASD. Responses of Yes (2 points), Maybe/A little (1 point), or No (0 points) had been checked for every item (total scores range 0–24). In this study, dichotomized scores (Yes and Maybe/A little = 1, No = 0) were used (total scores possible 0–12).

#### Regulatory Problems

RPs were defined as persistent problems with eating, sleeping, and/or excessive crying after the age of three months and regarded as serious problems by the caregiver and/or the clinician recorded RPs at the CHC and/or during the neuropsychiatric assessment. Based on onset age for symptoms, a division was made into three groups: very early onset (0–3 months), early onset (4–12 months), and later onset (13–18 months).

#### Body Mass Index and Growth Charts

In Sweden, growth charts are used with SD curves with growth lines for the mean and 1, 2, and 3 SDs below and above the mean (60). These have been used to describe growth and BMI in the study. The SD curve lines can also be understood as percentile lines with the following cumulative distribution: −3 SD: 0.1%, −2 SD: 2.3%, −1 SD: 15.9%, M: 50.0%, 1 SD: 84.1%, 2 SD: 97.7%, and 3 SD: 99.9%.

#### Medical Assessments, Including Laboratory Analyses

Analyzed data included broad medical assessments by a pediatrician and data from referral to other medical specialities when indicated. Blood tests, including hemoglobin and iron status, transglutaminase IgA antibodies, Vitamin D, and SNP microarray, had been analyzed for all children. In cases where there was a suspicion of food allergy/sensitivity based on symptoms and pediatrician clinical judgment, lab tests, and elimination and provocation tests, had been conducted.

### Classification and Definitions of Feeding Problems

**Figure 1:**
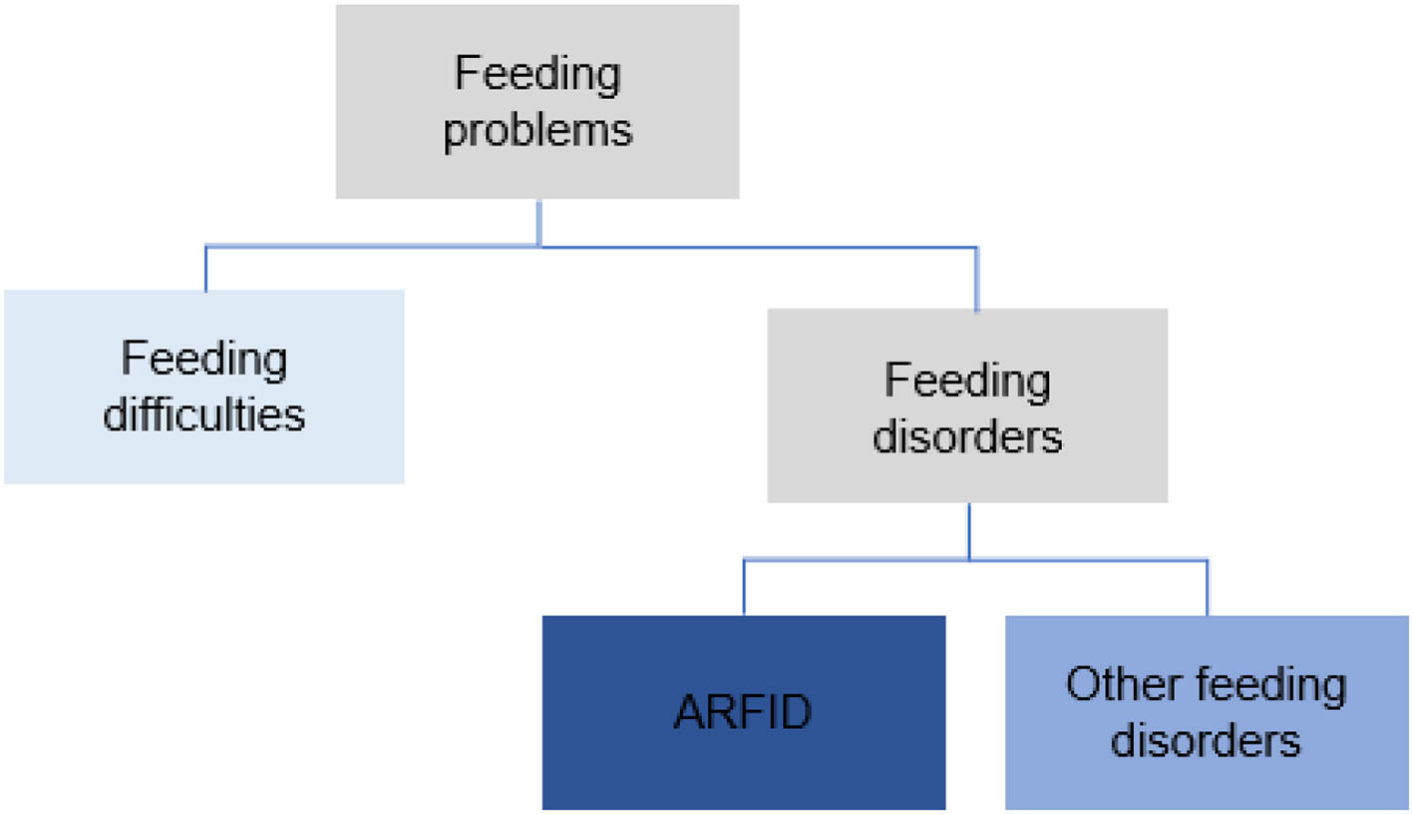
Classification of feeding problems.

The term feeding problems was used to cover all forms of problems with eating and feeding. These were divided into *feeding difficulties* and *feeding disorders* (Figure 1). Problems were classified as *feeding difficulties* when there was some parental concern regarding the child's eating behavior (some selectivity, mealtime behavior problems, or other transient problems not requiring treatment efforts from health care). *Feeding disorders* were defined as concerns regarding compromised growth and/or nutritional deficiency and/or difficulties during mealtimes persisting more than three months and requiring special efforts from health care; comprehensive assessments/treatments and interventions by a pediatrician (and when indicated other disciplines) and, e.g., a dietician, a psychologist or a speech and language therapist. *Onset age* for feeding problems was divided into four groups: 0–5 months, 6–12 months, 13–24 months, and >24 months. The feeding problems were studied retrospectively from the child's first months, and the classification and diagnosis of feeding disorders, including *ARFID*, was made at the time of the ASD diagnosis. Feeding disorders not meeting the criteria for ARFID were referred to as *other feeding disorders*. A second evaluation regarding feeding was made based on medical records two years after the first ASD diagnosis. *The DSM-5 criteria for ARFID* were used in the diagnostic process for all participants (40). For a diagnosis of ARFID, at least one criterion A1-A3 had to be met (only A4 not sufficient), as well as criteria B-D. Operationalizations for DSM-5 criteria A1-A4 were developed for this study (Supplement Material 2). The proposed criteria for *PFD* by Goday et al. (37) were also studied and used in the retrospective analyses for all children with feeding disorders (Supplement Material 3). The definition of the chronic form of the PFD (duration ≥ three months) was used.

### Data Analysis

Descriptive statistics were performed to provide a picture of the feeding problems and interventions. To test associations between two binary variables, Fisher's exact tests and risk ratios (RRs) were computed. To test associations between ordered categorical and binary variables, Kruskal-Wallis tests were used. Welch's *t*-test was used to test group differences for continuous variables. All tests were two-tailed and conducted at a 5% significance level. Computations were performed in Stata 16.1 (61).

## Results

### Distribution of Feeding Problems and Disorders

**Figure 2:**
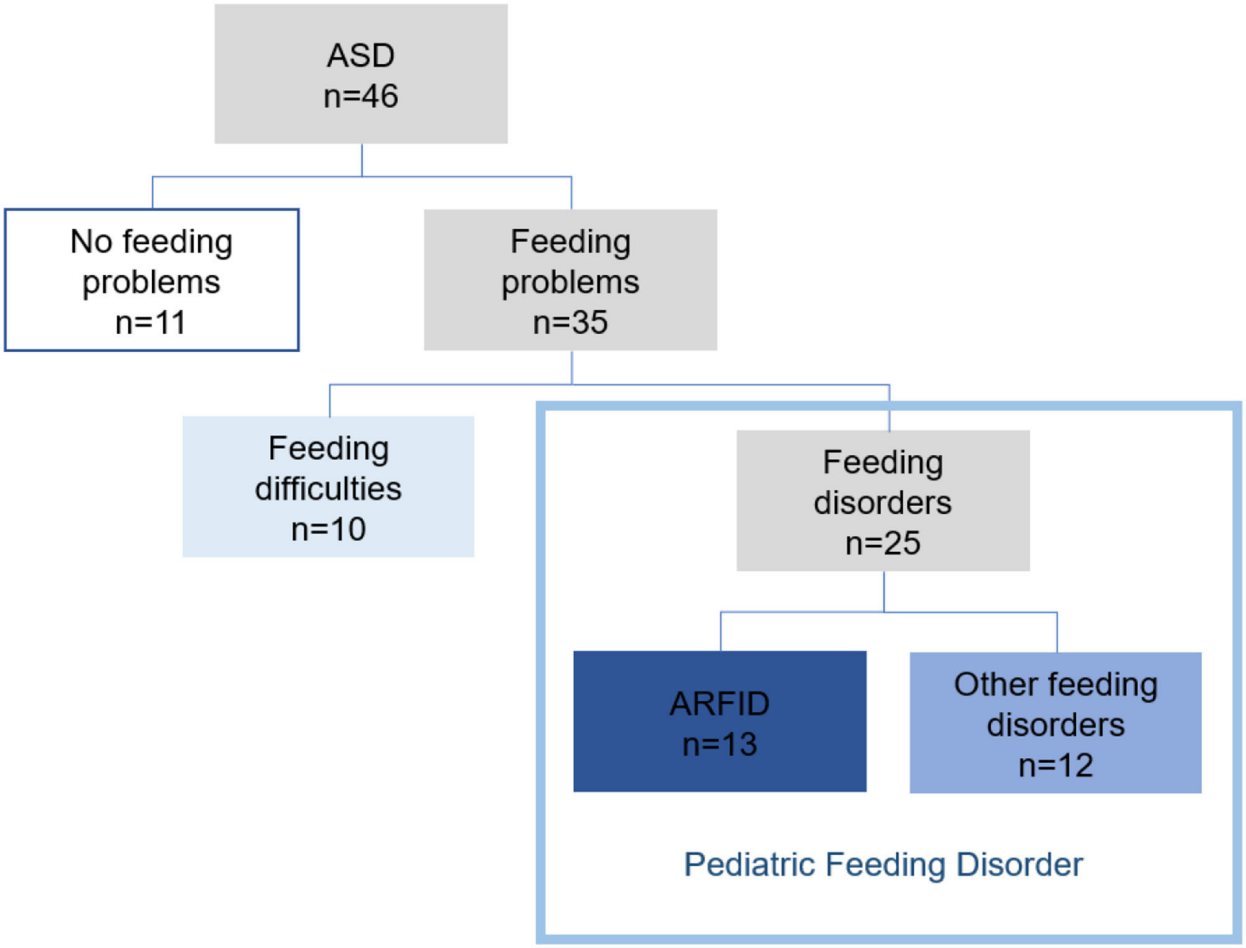
Feeding problems in children with ASD (*n* = 46).

For 11 (24%) of the children, there was no concern regarding feeding. Feeding problems were found in 35 (76%) of the 46 children. Feeding difficulties (some selectivity, mealtime behavior problems, or other problems not requiring treatment efforts from health care) were found in 10 (22%) children. In 25 children (54%), six girls and 19 boys, the feeding problems met the used definition of a feeding disorder. ARFID criteria were met in 13 (28%) and 12 (26%) children had other feeding disorders. Proposed criteria for PFD were met in all 25 children with feeding disorders (Figure 2).

### Clinical Characteristics of Children With vs. Without Feeding Problems

Most of the 46 children (89%) had been born at term, with no significant difference between the children with and without feeding problems (*p* = 0.317). Mean birth weight for children with feeding problems was lower (*M* = 3,142 g, range = 1,690–4,280 g, SD = 604), vs. (*M* = 3,835 g, range = 3,150–4, 790 g, SD = 427) (*t* = 4.22(23.8), *p* <0.001). The prevalence of birth complications (*p* = 0.441) and medical conditions during the neonatal period (*p* = 1) were comparable for the two groups (Table 1). The cognitive level did not differ between children with and without feeding problems (*p* = 0.767). Of the 35 children with feeding problems, 15 (43%) had ASD level 1, and 20 (57%) had ASD level 2. Among children without feeding problems, 10 (91%) had ASD level 1, and one child (9%) had ASD level 2. ASD level 2 was significantly more common in children with feeding problems (*p* = 0.006). Gender was equally distributed across the groups (*p* = 1).

**Table 1:** Comparison of participants characteristics including gender, perinatal risk factors, cognitive level, ASD level, and coexisting neurodevelopmental conditions (*n* = 46).

	**Total sample (*n =* 46)**	**Children with feeding problems** **(*n =* 35)**	**Children with no feeding problems** **(*n =* 11)**	***p* (2-sided)^a^**
	***n* (%)**	***n* (%)**	***n* (%)**	
**Gender**				
Male	37 (80.4)	28 (80.0)	9 (81.8)	1
Female	9 (19.1)	7 (20.0)	2 (18.2)	
**Perinatal risk factors**				
Clustering of birth complications^b^	12 (26.1)	8 (22.9)	4 (36.4)	0.441
Preterm birth^c^	5 (10.1)	5 (14.3)	0 (0)	0.317
Small size for gestational age (SGA)^d^	4 (8.7)	4 (11.4)	0 (0)	0.559
Other medical conditions during the neonatal period^e^	6 (13.0)	5 (14.3)	1 (9.1)	1
**Cognitive level** ^f^				
AIF	6 (13.0)	5 (14.3)	1 (9.1)	0.767^g^
BIF	12 (26.1)	8 (22.9)	4 (36.4)	
ID	28 (60.9)	22 (62.9)	6 (54.5)	
**ASD level**				
Level 1	25 (54.3)	15 (42.9)	10 (90.9)	0.006
Level 2	21 (45.7)	20 (57.1)	1 (9.1)	
Coexisting neurodevelopmental conditions^h^	46 (100.0)	35 (100.0)	11 (100.0)	–

a *Fisher's exact test was used except where otherwise specified. The test was conducted at a 5% significance level*.

b *Such as intrauterine hypoxia or birth asphyxia, including 5 min Apgar scores <7; elective and emergency cesarean section; and assisted birth, including vacuum extraction and forceps*.

c *Gestational age ≤ 36 weeks*.

d *>2 SD below the mean birth weight for the gestational age according to Swedish birth weight standards*.

e *Such as infections and neonatal jaundice*.

f *AIF, average intellectual functioning; BIF, borderline intellectual functioning; ID, intellectual disability*.

g *Kruskal-Wallis test was used*.

h *Such as sleeping problems, early ADHD symptoms, language disorders and/or epilepsy*.

ESSENCE-Q scores differed across groups. The average total scores for those with and without feeding problems were 13.9 (range = 9–22, SD = 3.1) and 11.5 (range = 7–16, SD = 2.7) (*t* = 2.3, *p* = 0.024, Cohen's *d* = 0.81, 95% CI 0.1–1.5). According to ESSENCE-Q-results, children with feeding problems more often had problems with sensory reactions (*p* = 0.032).

### Clinical Characteristics of Children With vs. Without ARFID

#### ARFID

According to retrospective analyses of all available data, 28% of the children (four girls and nine boys) fulfilled the ARFID criteria. The criteria were met at the time of ASD diagnosis in all but two of the 13 children. The *onset age* for feeding problems in ARFID was very early, before clinical suspicion of ASD. In nine children with ARFID, feeding problems had started during the child's first year, and in seven of them already during the first six months. All 13 children with ARFID had an apparent lack of interest in eating or in food and also avoidance based on sensory characteristics of food. At the time of ASD diagnosis, 10 children (77%) had a BMI ≤ −2 SD (A1). In two children in which ARFID developed later, a growth deviation (A1) was observed within one year after ASD diagnosis. Three children (23%) had been diagnosed with iron deficiency in need of treatment (A2). Eleven children (85%) had met a dietician, and nine of them (69%) had been prescribed oral nutritional supplements (A3). In 11 children (85%), the feeding behavior had marked interference on the child's psychosocial function (A4). Five children with ARFID also had one or more known coexisting medical conditions related to feeding, such as food allergy, adenoid hypertrophy (affecting both sleep and feeding and in need of surgery), oral motor difficulties, and/or operated cleft palate. In these children, the feeding problems met the criteria for ARFID, and the severity of the feeding disorder exceeded what could be expected from the coexisting medical condition related to feeding. Here, this subgroup is referred to as ARFID+ (Table 2).

**Table 2:** Clinical characteristics for ARFID in children with ASD including onset age for feeding problems, the start of treatment efforts for feeding problems in specialist health care (pre ASD diagnosis), and ARFID status at follow-up (*n* = 13).

**ID**	**Gender male/** **female**	**Onset age feeding problems (months)**	**Treatment efforts for feeding problems in specialist health care (pre ASD diagnosis) Age (months)**	**ASD diagnosis Age (months)**	**BMI in SD (at the time for ASD diagnosis)**	**Met ARFID criteria A^a^**				**Laboratory data**	**ARFID+^f^**	**Remission of ARFID at follow-up^g^**
						**A1^b^**	**A2^c^**	**A3^d^**	**A4^e^**			
1^h^.	M	0–5	–	24	0	1	0	0	1	0	1	Yes
2.	M	0–5	Newborn	27	≤ -2	1	1	1	1	Iron deficiency	1	No
3.	M	0–5	–	31	≤ -2	1	1	1	1	0	1	No
4.	F	0–5	–	32	≤ -2	1	0	0	1	0	0	Yes
5.	F	0–5	34	35	≤ -3	1	1	1	1	0	1	No
6.	F	0–5	22	47	≤ -2	1	1	1	1	0	0	Yes
7.	F	0–5	16	50	≤ -2	1	1	1	0	0	0	No
8.	M	6–12	20	33	≤ -2	1	1	1	1	0	1	No
9.	M	6–12	–	44	≤ -2	1	1	1	1	0	0	No
10.	M	13–24	–	29	≤ -3	1	0	0	1	0	0	No
11.	M	13–24	–	35	0	0	1	0	0	Iron deficiency	0	Yes
12.	M	13–24	38	39	≤ -2	1	1	1	1	0	0	No
13^h^.	M	>24	–	31	+1	1	1	1	1	Iron deficiency	0	No

a *The criteria B, C and D were met for all children*.

b *Significant weight loss (or failure to achieve expected weight gain or faltering growth in children)*.

c *Significant nutritional deficiency*.

d *Dependence on enteral feeding or oral nutritional supplements*.

e *Marked interference with psychosocial functioning*.

f *ARFID and coexisting medical conditions related to feeding problems*.

g *Follow-up, two years after ASD diagnosis*.

h *For this child, the criteria for ARFID, including growth deviations, were met in one year after the ASD diagnosis*.

**Figure 3:**
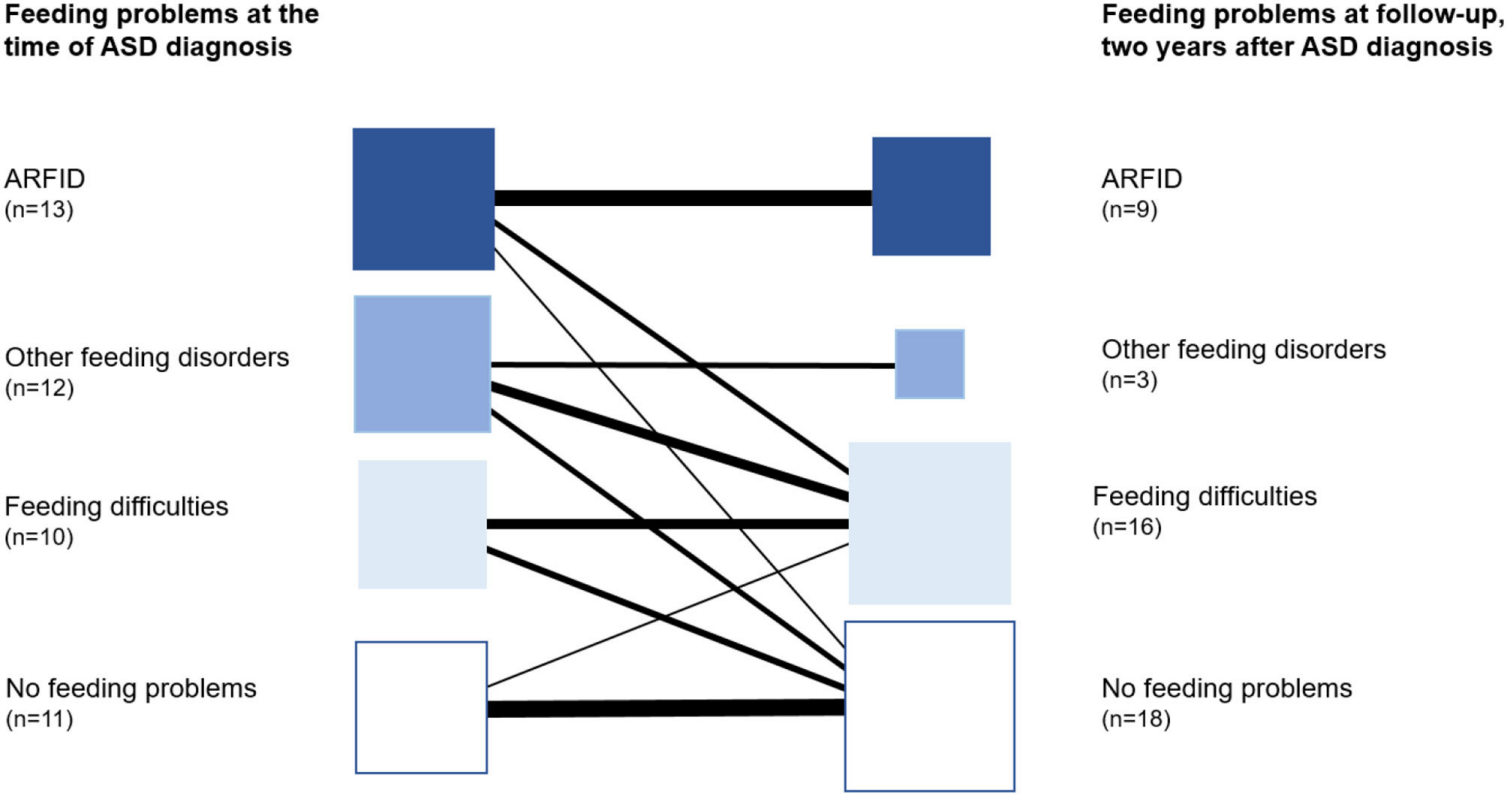
Course of feeding problems/disorders over two years (*n* = 46).

*Remission of ARFID* was found in four of the 13 children at follow-up two years after ASD diagnosis. In three of them, some feeding difficulties still existed but not meeting the criteria for ARFID, and in one child, no feeding difficulties were found (Figure 3).

#### Other Feeding Disorders

Twelve children with ASD (26%) had *other feeding disorders than ARFID*, and in four of these, the feeding problems had started during the child's first year. In this group, lack of interest in eating or food and/or a selectivity was found in eight children, but not to the extent that the criteria for ARFID were met. Seven children had one or more known medical conditions related to the feeding disorder, including multiple food allergies, tonsil, or/and adenoid hypertrophy. At the time of the diagnosis of ASD, only 1/12 children in this group had a BMI ≤ 2. One child was treated for iron deficiency. Dieticians had assessed five children due to selective eating or multiple food allergies, but none had been prescribed oral nutritional supplements. At follow-up two years after the first ASD diagnosis, nine children did not meet the criteria for a feeding disorder anymore. Six of these were found to have some feeding difficulties (Figure 3).

#### PFD

All 25 children with feeding disorders met the proposed criteria for *PFD* (Figure 2, Supplementary Material 3). According to the PFD criteria, none of the children had any difficulties in the domain medical dysfunction (A.1). In 14 children, of whom 12 were children with ARFID, one or more criteria for nutritional dysfunction (A.2) were met. In the domain feeding skill dysfunction, six children needed texture modification of liquid or food (A.3a). Most children (96%) met at least one criterion for psychosocial dysfunction (A.4).

### Early Symptoms, Prodromal Signs for Feeding Disorders

Of the 46 children in the study group, 22 (48%) had persisting RPs after three months of age, and the onset age of RPs was very early. RP presence significantly increased the risk of later feeding disorder (RR = 2.32; 95% CI 1.26–4.26). Particularly RPs concerning eating (little appetite, swallowing problems, vomiting, and/or problems with mealtime routines) significantly increased the risk for later feeding disorder (RR = 2.11; 95% CI 1.32–3.38) (Table 3).

**Table 3:** Association between persisting regulatory problems (RPs) and having a feeding disorder vs. feeding difficulties/no feeding problems in children with ASD (*n* = 46).

	**Total sample** **(*n =* 46)**	**Children with feeding disorders** **(*n =* 25)**	**Children with feeding difficulties or no feeding problems** **(*n =* 21)**	**Risk ratio (95% CI)**	**p (2-sided)^a^**
	***n* (%)**	***n* (%)**	***n* (%)**		
**Any RP**	22 (47.8)	17 (68.0)	5 (23.8)	2.32 (1.26, 4.26)	0.004
**Type of RP**					
Eating	14 (30.4)	12 (48.0)	2 (9.5)	2.11 (1.32, 3.38)	0.009
Sleeping	16 (34.8)	12 (48.0)	4 (19.0)	1.73 (1.05, 2.85)	0.063
Excessive crying	13 (28.3)	9 (36.0)	4 (19.0)	1.42 (0.86, 2.37)	0.325
**Age of RP onset**					0.285^b^
Very early onset (0–3 months)	17 (37.0)	14 (82.4)	3 (60.0)	–	–
Early onset (4–12 months)	3 (6.5)	2 (11.8)	1 (20.0)	–	–
Late onset (13–18 months)	2 (4.3)	1 (5.9)	1 (20.0)	–	–

a *Fisher's exact test was used except where otherwise specified*.

b *Kruskal-Wallis test was used*.

*All test was conducted at a 5% significance level*.

## Discussion

Frequent feeding problems were found in these preschool children with ASD (76%). While one in five (22%) had some “non-disorder level” *feeding difficulties*, more than half (54%) had a *feeding disorder* (ARFID or other feeding disorder) requiring specific health care services, including multidisciplinary assessments and treatment. Only about one in four children with ASD showed no problem with feeding.

The prevalence of ARFID in preschool children with ASD was high (28%). All children in the ARFID group had an apparent lack of interest in eating or food and avoidance based on sensory characteristics of food. However, some degree of selectivity and/or lack of interest in eating or food were the most common symptoms in all children with feeding problems, not just in children with ARFID. In feeding problems meeting the ARFID criteria, the problems were severe and persisting. Children with ARFID had lower BMI at the age of ASD diagnosis compared to those with other feeding disorders. Most children with ARFID needed nutritional supplements for long periods. At follow-up two years after ASD diagnosis, criteria for ARFID were still met in 69% of the children compared with declining symptoms and more common remission in children with other feeding problems (Figure 3). The remission we found in children with feeding problems other than ARFID may have several individual explanations. There is a need for future research to understand the covariates and factors involved in feeding problems and the persistence of these. For some children in our study, the problems might have been the same transient nature as reported in a general child population (62). We also propose that the treatment efforts in the multiprofessional team not just for core symptoms in ASD but for the individual child's needs (other neurodevelopmental and behavioral problems, medical conditions) and the family's needs are essential. However, the same remission was not found in children with ARFID. Interestingly, a recent study of the developmental progression of feeding problems in preschool children with ASD showed that feeding problems remitted over time in most children, while a small subgroup showed chronic feeding problems into school age (63).

The diagnostic process in ARFID, including differential diagnostics, is challenging. For research on AFRID in general and prevalence estimates in particular, agreed definitions and threshold levels for meeting diagnostic criteria are essential. In order to make the present study possible, operationalizations for the DSM-5 ARFID criteria A1-A4 were developed. Clear definitions were needed for children in this age group, e.g., for weight loss or failure to achieve appropriate weight gain (A2). The DSM-5 ARFID criteria were interpreted relatively strictly (40). Meeting only criterion A4 (marked interference with psychosocial functioning), but not criteria A1-A3, was not sufficient for diagnosis in children with avoidant/restrictive eating; that is, in all children diagnosed with ARFID, at least one of the criteria A1-A3 was met. Our study raises the question of whether it is possible to define interference with psychosocial functioning related to feeding in young children with ASD and other coexisting medical and/or neurodevelopmental conditions. When it comes to children living in a low resource area, this might be even more difficult. However, we also found that thanks to caregivers' creative solutions and collaboration with preschool staff, some children clinically at great risk of fitting ARFID criteria still had a sufficient nutritional intake not meeting the ARFID criteria. The challenge in differential diagnosis and the need to consider developmental stage and context of feeding when considering ARFID diagnosis has recently been highlighted by the ARFID workgroup (54). The workgroup proposed that diverse developmental manifestations of ARFID criteria may need to be added.

Our ARFID prevalence (28%) is higher than reported in a general population of 4–7-year old children (1.3%) (53) and school-aged children (0.3–5.5%) (49, 50, 52). However, there is to our knowledge, no previously reported estimates of ARFID prevalence in preschool children with ASD based on clinical assessments. Although our study group from a multiethnic low-resource area was small and might not be representative for all children with ASD, together with earlier research reporting frequent feeding problems in children with ASD (1, 2) and the experiences from clinicians in the field, our results indicate that ARFID is a common coexisting problem in children with ASD. Another current study also highlights the high comorbidity of ARFID in ASD with an estimated ARFID prevalence of 21% based on parent questionnaires from a large autism cohort (64).

Findings from this study also provide support for the use of the recently proposed broad criteria for PFD (37). The PFD criteria were met for all children with a feeding disorder, including those with ARFID and other feeding disorders. The proposed concept of PFD covering different domains might promote and clarify the need for a broad approach both in assessments and treatment efforts for children with feeding disorders. However, more clarity is needed regarding categorizations and diagnostic classifications of feeding problems in research and clinical practice.

Persistent RPs have been pointed out as red flags for later developmental disorders (15). However, few studies have specifically focused on a possible link between persisting RPs and early presentations of ASD. Schmid et al. (65) found that RPs in general, particularly early feeding problems, predicted deficits in social skills in preschool children. Barnevik Olsson et al. (12) reported that the odds for children with ASD to have early consultations at the CHC related to RPs was four times higher than for children in a comparison group. In our study, we found that almost 50% of the children had persisting RPs before ASD diagnosis, indicating a much higher prevalence of RPs than the reported prevalence of 9% in a general population (16). Particularly eating-related RPs were found to increase the risk of later feeding disorders. We suggest that persisting RPs, in general, might be prodromal signs for ASD, and especially eating-related RPs may be early signals predicting later coexisting feeding disorders in ASD. Persisting RPs, but also early unusual sensory reactions, especially regarding taste and smell, should alert the clinician to consider ASD and feeding problems (8, 66). In our study, parents of children with ASD and feeding problems reported unusual sensory reactions, and these children had higher sensory reactions scores on the ESSENCE-Q.

Our results have implications for health care staff, including pediatricians and other clinicians assessing infants. Children with persisting RPs, unusual sensory reactions, early feeding problems, early social communications deficits, or any other early neurodevelopmental symptom (ESSENCE) need to be followed up and broadly assessed. Different professionals need to work closely together to promote important assessments and treatment efforts, such as considering early symptoms of ASD in an infant with feeding problems or the need for medical assessments in children with ASD and coexisting conditions.

## Data Availability Statement

The datasets presented in this article are not readily available because the study is ongoing; therefore, the research data are not shared. Requests to access the datasets should be directed to petra.linnsand@vgregion.se.

## Ethics Statement

The studies involving human participants were reviewed and approved by The Regional Ethics Committee in Gothenburg, Sweden, Registration 653-14. Written informed consent to participate in this study was provided by the participants' legal guardian/next of kin.

## Informed Consent

All parents provided verbal and written informed consent to participate in the study. Informed consent was obtained from at least one parent of each child.

## Author Contributions

GN and PL were part of the study design development, data collection, data analysis and writing. MJ was part of the study design development. JH and LD were part of the statistical analysis and interpretation of the data. CG was part of the study design development and writing. All authors provided a critical review of the manuscript and approved the final draft.

## Funding

This study was supported by Grants from the R&D Committee at Angered Hospital/SV Hospital group, Western Gotaland County Council, and the Kempe-Carlgrenska Foundation, Sweden. Open access is funding provided by the University of Gothenburg.

## Conflict of Interest

The authors declare that the research was conducted in the absence of any commercial or financial relationships that could be construed as a potential conflict of interest.

## Publisher's Note

All claims expressed in this article are solely those of the authors and do not necessarily represent those of their affiliated organizations, or those of the publisher, the editors and the reviewers. Any product that may be evaluated in this article, or claim that may be made by its manufacturer, is not guaranteed or endorsed by the publisher.

## Abbreviations

ASD: Autism Spectrum Disorder
ARFID: Avoidant Restrictive Food Intake Disorder
PFD: Pediatric Feeding Disorder
RPs: Regulatory Problems
ESSENCE: Early Symptomatic Syndromes Eliciting Neurodevelopmental Clinical Examinations
CHC: Child Health Center.
