# Supplementary material for: Feeding Problems Including Avoidant Restrictive Food Intake Disorder in Young Children With Autism Spectrum Disorder in a Multiethnic Population
Source: Front Pediatr. 2021 Dec 13;9:780680. doi: 10.3389/fped.2021.780680 (PMC8710696; doi:10.3389/fped.2021.780680)
Supplement: Supplementary file 1 [file Data_Sheet_1.PDF]

Supplement 1 ESSENCE Questionnaire

ESSENCE-Q-REV (Gillberg C 2012)

Name of child:	

Age:		Completed by:	
Sex:		Date:	

Please take a few minutes to read and check the following items.

- Y= Yes
- M/AL = Maybe/A little
- N= No

Have you (or anybody else, who?_______________) been concerned for more than a few months regarding child´s

1. General development ☐
2. Motor development/ milestones ☐
3. Sensory reactions (e.g. touch, sound, light, smell, taste, heat, cold, pain) ☐
4. Communication/language/ babble ☐
5. Activity (overactivity/passivity) or impulsivity ☐
6. Attention/concentration/ "listening" ☐
7. Social interaction/interest in other children ☐
8. Behaviour (e.g. repetitive, routine insistence) ☐
9. Mood (depressed, elated/manic, extreme irritability, crying spells) ☐
10. Sleep ☐
11. Feeding ☐
12. "Funny spells" / absences ☐

If Y or M/AL to any of the above, please elaborate briefly here:
