# Supplementary material for: Feeding Problems Including Avoidant Restrictive Food Intake Disorder in Young Children With Autism Spectrum Disorder in a Multiethnic Population
Source: Front Pediatr. 2021 Dec 13;9:780680. doi: 10.3389/fped.2021.780680 (PMC8710696; doi:10.3389/fped.2021.780680)
Supplement: Supplementary file 2 [file Data_Sheet_2.PDF]

Supplement 2 Operationalizations for the DSM-5 ARFID criteria A1-4 used in the present study

Criterion A1 - Significant weight loss (or failure to achieve expected weight gain or faltering growth in children): The A1 criterion was defined as BMI ≤ -2SD for more than three months or growth delay defined as a deflection of >0.5 SD in weight and height per year in growth charts or a deflection of >1 SD at any time at the age of 2-6 years.

Criterion A2 - Significant nutritional deficiency: The A2 criterion was defined as deviations in laboratory data (iron deficiency, S-Fe <µg15 and/or low 25(OH)D-Vitamin <30nmol/ in need of treatment and/or insufficient nutritional intake according to diarized daily logs (according to dietician assessment).

Criterion A3 - Dependence on enteral feeding or oral nutritional supplements: The A3 criterion was defined as dependence on enteral feeding or oral nutritional supplements after dietician assessment ≥ 1 supplement drink (300-400 kcal) per day.

Criterion A4 - Marked interference with psychosocial functioning: The A4 criterion was defined as an inability to attend preschool or ability to attend only for 3 hours per day due to inability to manage eating situation at preschool and/or marked fatigue/irritability reported due to nutritional deficiency at preschool and/or at home.
