# Supplementary material for: Feeding Problems Including Avoidant Restrictive Food Intake Disorder in Young Children With Autism Spectrum Disorder in a Multiethnic Population
Source: Front Pediatr. 2021 Dec 13;9:780680. doi: 10.3389/fped.2021.780680 (PMC8710696; doi:10.3389/fped.2021.780680)
Supplement: Supplementary file 3 [file Data_Sheet_3.PDF]

Supplement 3 Diagnostic criteria for PFD proposed by Goday et al. (37)

A. A disturbance in oral intake of nutrients, inappropriate for age, lasting at least 2 weeks and associated with 1 or more of the following:

1. Medical dysfunction, as evidenced by any of the following
    a. Cardiorespiratory compromise during oral feeding
    b. Aspiration or recurrent aspiration pneumonitis
2. Nutritional dysfunction, as evidenced by any of the following:
    a. Malnutrition
    b. Specific nutrient deficiency or significantly restricted intake of one or more nutrients resulting from decreased dietary diversity
    c. Reliance on enteral feeds or oral supplements to sustain nutrition and/or hydration
3. Feeding skill dysfunction, as evidenced by any of the following:
    a. Need for texture modification of liquid or food
    b. Use of modified feeding position or equipment
    c. Use of modified feeding strategies
4. Psychosocial dysfunction, as evidenced by any of the following:
    a. Active or passive avoidance behaviors by child when feeding or being fed
    b. Inappropriate caregiver management of child's feeding and/or nutrition needs
    c. Disruption of social functioning within a feeding context
    d. Disruption of caregiver-child relationship associated with feeding

B. Absence of the cognitive processes consistent with eating disorders and pattern of oral intake is not due to a lack of food or congruent with cultural norms.
